# Lure-and-Kill Yeast Interfering RNA Larvicides Targeting Neural Genes in the Human Disease Vector Mosquito *Aedes aegypti*

**DOI:** 10.1038/s41598-017-13566-y

**Published:** 2017-10-16

**Authors:** Limb K. Hapairai, Keshava Mysore, Yingying Chen, Elizabeth I. Harper, Max P. Scheel, Alexandra M. Lesnik, Longhua Sun, David W. Severson, Na Wei, Molly Duman-Scheel

**Affiliations:** 1 Indiana University School of Medicine, Department of Medical and Molecular Genetics, South Bend, IN USA; 2 The University of Notre Dame Eck Institute for Global Health, Notre Dame, IN USA; 3 The University of Notre Dame Department of Biological Sciences, Notre Dame, IN USA; 4 The University of Notre Dame Department of Civil and Environmental Engineering, Notre Dame, IN USA

## Abstract

New mosquito control strategies are vitally needed to address established arthropod-borne infectious diseases such as dengue and yellow fever and emerging diseases such as Zika and chikungunya, all of which are transmitted by the disease vector mosquito *Aedes aegypti*. In this investigation, *Saccharomyces cerevisiae* (baker’s yeast) was engineered to produce short hairpin RNAs (shRNAs) corresponding to the *Aedes aegypti* orthologs of *fasciculation and elongation protein zeta 2* (*fez2*) and *leukocyte receptor cluster* (*lrc*) member, two genes identified in a recent screen for *A*. *aegypti* larval lethal genes. Feeding *A*. *aegypti* with the engineered yeasts resulted in silenced target gene expression, disrupted neural development, and highly significant larval mortality. Larvicidal activities were retained following heat inactivation and drying of the yeast into tabular formulations that induced >95% mortality and were found to attract adult females to oviposit. These ready-to-use inactivated yeast interfering RNA tablets may one day facilitate the seamless integration of this new class of lure-and-kill species-specific biorational mosquito larvicides into integrated mosquito control programs.

## Introduction

Larviciding is a key component of integrated control and disease prevention strategies targeting *Aedes* mosquitoes, which breed in water-filled containers located within or close to human dwellings^1^. Given the increase of reported insecticide resistance and rising concern for the negative effects of pesticides on non-target organisms, the current pesticide repertoire is faced with great challenges to sustainability. Due to the lack of vaccines, mosquito control is the primary means of disease control, and new biorational pesticides are vitally needed to address established arthropod-borne diseases such as dengue and emerging infectious diseases such as Zika. Although it is beginning to attract attention in agricultural biotechnology communities^2^, the use of RNA interference (RNAi) is a largely unexplored approach for control of disease vector mosquitoes. While most mosquito researchers use longer (300-400 bp) double stranded RNA (dsRNA) molecules for RNAi, the short length (21-25 bp) of custom small interfering RNAs (siRNAs) facilitates the design of interfering RNA that recognizes genes in targeted organisms, but not in non-target species. We have demonstrated that ingested siRNAs can be used to selectively target *Aedes* larval genes and have used this technology to characterize mosquito larval development^3–8^. These siRNAs and their short hairpin RNA (shRNA) counterparts may represent a novel class of larvicides with untapped potential for sustainable mosquito control.

Two siRNA molecules, #52 and #101, which correspond to the *Aedes aegypti fasciculation and elongation zeta 2* (*fez2*) and the receptor-encoding ortholog of *leukocyte receptor cluster* (*lrc*) member genes, respectively, were identified in recent screens for mosquito larval lethal genes, which will be described in their entirety elsewhere. These two interfering RNAs were prioritized, due to their high larvicidal activities and lack of conserved target sites in humans and other non-target organisms, for further characterization. This research investigation examines the hypothesis that *Saccharomyces cerevisiae* (baker’s yeast) genetically engineered to express interfering RNAs #52 and #101 can function as *A*. *aegypti* larvicides. The use of RNAi for mosquito control requires effective and affordable methods of RNA production and delivery. Yeast can be genetically engineered to produce larvicidal interfering RNA, which is then propagated at affordable costs through cultivation of the bioengineered yeast. Yeast is known to function as an oviposition attractant for gravid adult females^9,10^, and yeast interfering RNA larvicides could therefore serve as a lure to attract gravid females to lay eggs in treated containers, where their offspring will be killed. Moreover, *S*. *cerevisiae* is a source of nutrition and a strong odorant attractant that can act as a bait^5^ to lure and then kill *A*. *aegypti* larvae that hatch in the containers. The results of this investigation indicate that genetically engineered yeast expressing interfering RNA corresponding to mosquito genes required for larval viability can function as lure-and-kill mosquito larvicides.

## Results and Discussion

### S. cerevisiae expressing shRNA targeting larval lethal genes effectively kills *A. aegypti* larvae

**Figure 1:**
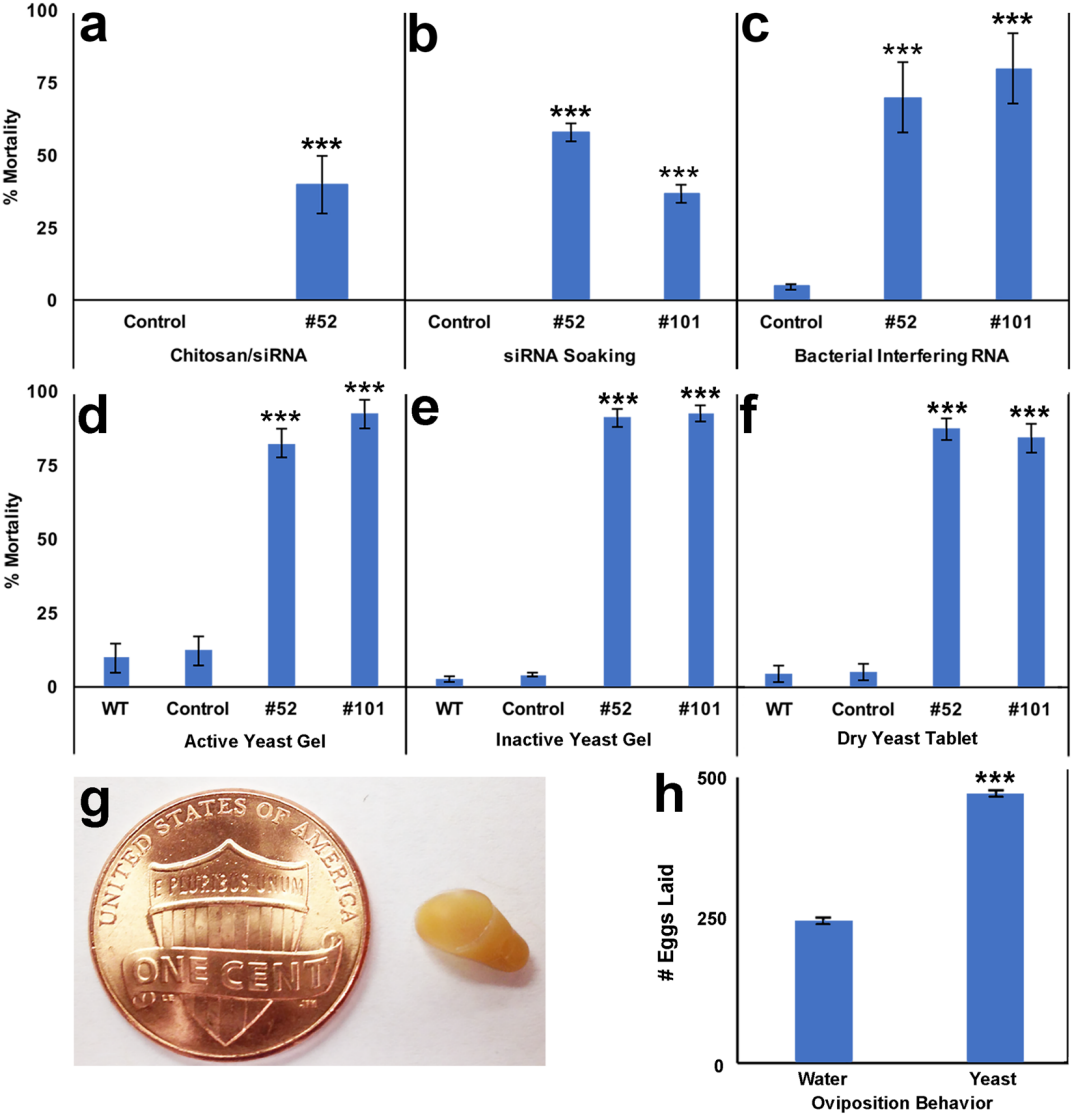
Larval mortality induced by interfering RNA larvicides #52 and #101. (**a**) siRNA #52 was identified in a chitosan nanoparticle larvicide screen in which it induced significant larval death; control larvae that ingested siRNA nanoparticles with no known *Aedes* target site survived. The screen was performed in duplicate (10 animals/treatment). (**b**) siRNA larvicide #101 was identified in a soaking screen. Although control siRNA-treated animals lived, significant larval mortality was observed in larvae soaked in siRNA #101 or #52; experiments were performed in triplicate with 20 animals/treatment and evaluated with Fisher’s exact test. (**c**) Significant mortality was observed in larvae fed with heat-inactivated bacteria expressing dsRNA corresponding to the siRNA #52 or #101 target sites, while animals fed a normal lab diet (WT) or control dsRNA bacteria survived (results were compiled from seven replicate experiments with 20 animals/treatment and assessed by ANOVA with Tukey’s multiple comparison test). Significant larval death was observed in larvae fed with gel-coated active (**d**), gel-coated heat-inactivated (**e**), or heat-inactivated dried (**f**) yeast larvicides #52 and #101; WT larvae or larvae fed yeast expressing control shRNA survive. No significant differences were observed in larvae fed a WT vs. control interfering RNA diet (**d-f**; not shown in **a-c**) or in the larvicidal capacity of active vs. heat-inactivated or gel-coated vs. dry yeast interfering RNA larvicides #52 or #101 (**d-f**). Results compiled from two (**d**) or three (**e,f**) biological replicates with four replicate containers of 20 larvae/replicate were analyzed by ANOVA with Tukey’s multiple comparison test. Ready-to-use dried and heat-inactivated yeast interfering RNA tablets (**g**, penny shown for scale) could be seamlessly integrated with current vector control strategies. (**h**) 10 gravid adult females deposited significantly more eggs in ovicups containing rain water with dried yeast interfering RNA tablets (470 ± 44 eggs laid) vs. rain water alone (248 ± 34 eggs laid); data (4 replications with 5 repetitions, n = 20) were analyzed with a paired t-test. Data are represented as mean % mortality, and error bars represent standard errors of the mean. ***P < 0.001 in comparison to control larvae (**a-f**) or vs. water alone (**h**); see text for exact P values.

siRNA #52, which corresponds to a target sequence in the first exon of *fez2* ^11^ (Supplementary Fig. S1a), was identified in a chitosan nanoparticle larvicide screen in which it induced 40 ± 10% larval death (Fig. 1a; P = 0.00327 vs. control siRNA treatment). Larvicidal activity of this interfering RNA was confirmed in soaking (Fig. 1b) and bacterial interfering RNA feeding experiments (Fig. 1c) in which it induced 58 ± 3% (P = 1.0 × 10^−5^ vs. control siRNA) and 70 ± 12% mortality (P = 0.0012 vs. control dsRNA bacteria treatment), respectively. siRNAs #414 and #418, which correspond to two alternative target sequences in *fez2*, were found to induce 47 ± 2% (P = 0.00611 vs. control siRNA) and 48 ± 3% (P = 0.006107 vs. control siRNA) mortality, respectively, in microinjection experiments, providing further evidence that *fez2* is a larval lethal gene in *A*. *aegypti*.

siRNA larvicide #101, which corresponds to a target sequence in the first exon of *lrc* ^11^ (Supplementary Fig. S1b), was identified in a larval soaking screen (37 ± 3% mortality observed; Fig. 1b; P = 1.0 × 10^−5^. vs. control siRNA treatment) and confirmed in bacterial interfering RNA feeding experiments (80 ± 12% mortality induced; Fig. 1c; P = 0.0003 vs. control dsRNA bacteria treatment). siRNAs #467 and #468, which correspond to alternative target sequences in *lrc*, were found to induce 38 ± 2% (P = 0.00440 vs. control siRNA) and 40 ± 2% (P = 0.01965 vs. control siRNA) mortality, respectively, in soaking experiments, providing further evidence that *lrc* is required for *A*. *aegypti* larval viability.

Given the significant larvicidal capacity of interfering RNAs #52 and #101 observed in conjunction with multiple delivery systems, these RNAs were selected for evaluation in a yeast delivery system. *Pichia pastoris* has been used for delivery of recombinant DNA and dsRNA to *Aedes* ^12,13^. *S*. *cerevisiae* was chosen for our investigation, as many genetic strains and plasmids are available for this genetic model organism. Furthermore, it is used very broadly for commercial applications, which might facilitate commercialization of this putative intervention in the future. shRNAs corresponding to the #52 and #101 target sequences were constitutively expressed from a non-integrating multi-copy yeast shuttle plasmid that was transformed into *S*. *cerevisiae*. These yeast strains, as well as control yeast expressing shRNA with no known target in *A*. *aegypti*, were fed to larvae in an agarose gel-covered formulation. While control-fed larvae survived (Fig. 1d,e), gel-coated live yeast expressing shRNA larvicides #52 and #101 killed 83 ± 7% (P = 0.0001 vs. control yeast interfering RNA treatment) and 93 ± 4% (P = 0.0001 vs. control yeast interfering RNA treatment) of mosquitoes, respectively (Fig. 1d). Longer pieces of dsRNA (~200 bp) targeting *Drosophila suzukii* genes have been used to silence target genes in *S*. *cerevisiae* ^14^. Our discovery that short hairpins with 25 bp target sequences can induce larval death is useful, as it facilitates the design of interfering RNA pesticides with little potential to affect non-target species.

Whyard *et al*.^15^ reported that dsRNA expressed in *E*. *coli* maintained its activity following heat-killing of the bacteria. The impact of heat-killing the yeast prior to delivery of shRNA larvicides, which is unknown, was therefore explored. Heat-inactivated gel-coated yeasts #52 and #101 induced 91 ± 3% (P = 0.0001 vs. control treatment) and 93 ± 3% (P = 0.0001 vs. control treatment) mortality, respectively (Fig. 1E). No significant differences were observed in the larvicidal capacity of live vs. heat-inactivated yeast interfering RNA larvicides (Fig. 1d,e). These results suggest that it will be possible to use heat-inactivated yeast interfering RNA larvicides, a finding that will greatly facilitate the potential use of these larvicides in the field, where the release of live yeast may not be desirable.

### Dry inactivated yeast tablets retain larvicidal activity and function as oviposition attractants

Although agarose-coated yeast larvicides induced high mortality rates (Fig. 1a,b), these wet and gummy formulations are not user-friendly. Moreover, water in containers treated with gel-coated yeast becomes cloudy, which could impact female oviposition behavior and would not be desirable for treatment of potable water. Dried tablets of inactivated *S*. *cerevisiae* (Fig. 1g) expressing shRNA (Fig. 1a), which are comparable in appearance to yeast nutritional tablet human dietary supplements, were prepared. This user-friendly formulation does not cloud the water and sinks to the bottom of treated containers, where *A*. *aegypti* larvae readily consume it. When used to treat 50 ml water in containers with 20 larvae, a 95 mg dried tablet of yeast larvicides #52 or #101 induced 88 ± 4% and 85 ± 5% larval mortality, respectively (Fig. 1f; P = 1.0 × 10^−5^). These tablets (LD50 #52 = 31.07 mg; LD50 #101 = 33.41 mg; Table 1a, Supplementary Fig. S2a,b), which effectively kill larvae in containers with larger volumes of water and varying larval densities (Table 1b), maintain significant residual activity after one week of submersion in water (Table 1c; P = 1.0 × 10^−5^).

**Table 1:** Analysis of heat-inactivated dried yeast pellet activity.

a. LD50 of dried yeast formulations						
Yeast	N	LD50 (mg)	95% confidence limit (mg)			
			Lower	Upper		
#52	45	31.07	27	35.03		
#101	33	33.41	28.73	38.04		
**b. Dried yeast formulation activity in containers with varying water volumes/larval densities**						
**Water volume (L)**	**Water depth (cm)**	**Number of larvae**	**Yeast (mg)**	**Yeast**	**Mean mortality induced**	**SEM**
0.050	1.7	20	85	Control	3.33%^a^	1.36%
				#52	86.67%^b^	1.57%
				#101	87.78%^b^	1.14%
1.50	2	100	425	Control	5.67%^c^	1.28%
				#52	83.67%^d^	1.41%
				#101	86.17%^d^	1.46%
15.75	26	80	340	Control	45.83%^e^	7.61%
				#52	95.63%^f^	2.14%
				#101	95.00%^f^	2.20%
**c. Residual activities of dried yeast formulations**						
**Yeast**	**Week 0**		**Week 1**		**Week 2**	
	**Mean mortality**	**SEM**	**Mean mortality**	**SEM**	**Mean mortality**	**SEM**
Control	6.11%^g^	1.72%	7.78%^i^	2.95%	6.27%^k^	2.94%
#52	90.00%^h^	1.11%	54.44%^j^	2.99%	7.78%^k^	2.24%
#101	93.33%^h^	1.57%	55.56%^j^	5.00%	13.33%^k^	3.77%

LD50 values and confidence intervals (a), larvicide activities in varying container sizes/larval densities (b), and larvicide residual activities (c) are shown for dried inactivated larvicide #52 and #101 yeast tablets vs. control interfering RNA tablets. For each experiment (treatments at the indicated water volumes/larval densities in b or residual activities at the indicated time points in c), means in the same column followed by different letters are significantly different (P = 1.0 × 10^−5^). No differences in mortality were noted between control interfering RNA yeast-fed animals (a-c) vs. animals fed a normal laboratory diet that were reared in parallel (not shown). No significant differences were detected between containers treated with larvicides #52 vs. #101 in a-c. SEM = standard error of the mean. Information concerning n numbers for each experiment is detailed in the methods section.

Active yeast, which releases carbon dioxide, is known to attract gravid female mosquitoes^9,10^, but the impact of dried inactivated yeast interfering RNA tablets on oviposition container choice is unknown. *A*. *aegypti* oviposition choice experiments demonstrated that in comparison to ovicups with water alone, gravid females deposit significantly more eggs in ovicups containing inactivated dry yeast tablets (Fig. 1h; P = 0.00131). These data suggest that inactivated dry yeast could be used as lure-and-kill larvicides that attract both *A*. *aegypti* larvae and gravid adult females.

### Yeast interfering RNA larvicides #52 and #101 silence target gene expression in the larval brain and disrupt neural synapses

The modes of action for larvicides #52 and #101 were next explored. No obvious external morphological defects or motor deficits were noted in larvicide-treated animals, which grew to normal size, as evidenced by the lack of body length differences in control vs. larvicide-treated animals (P = 0.116) in early L4, just prior to their death as fourth instar larvae (Supplementary Fig. S2c,d). The prospective target of #52, *fez2*, is orthologous to *unc-76*, an evolutionarily conserved cytosolic protein that binds to the kinesin heavy chain. In *D*. *melanogaster*, Unc-76 functions in kinesin-mediated transport in neurons. *Drosophila* loss-of-function mutants display neural defects and die prior to pupation^16^. The prospective target of #101, *lrc*, is an ortholog of *D*. *melanogaster CG6700*, which encodes a conserved protein containing a C-terminal SAC3/GANP domain that is a component of the Dystroglycan-Dystrophin (Dg-Dys) complex^17,18^ and is required for viability^19^. *CG6700* was recently included in a catalog of genes likely to function in synapse assembly and function^20^, although its role in this capacity remained to be tested. Given that the *Drosophila* orthologs of *fez2* and *lrc* have been implicated in fly neural development and function, the impact of silencing these genes during larval development was assessed in the *A*. *aegypti* larval nervous system during early L4 just prior to the time at which larvicide treated animals die (Supplementary Fig. S2c,d).

**Figure 2:**
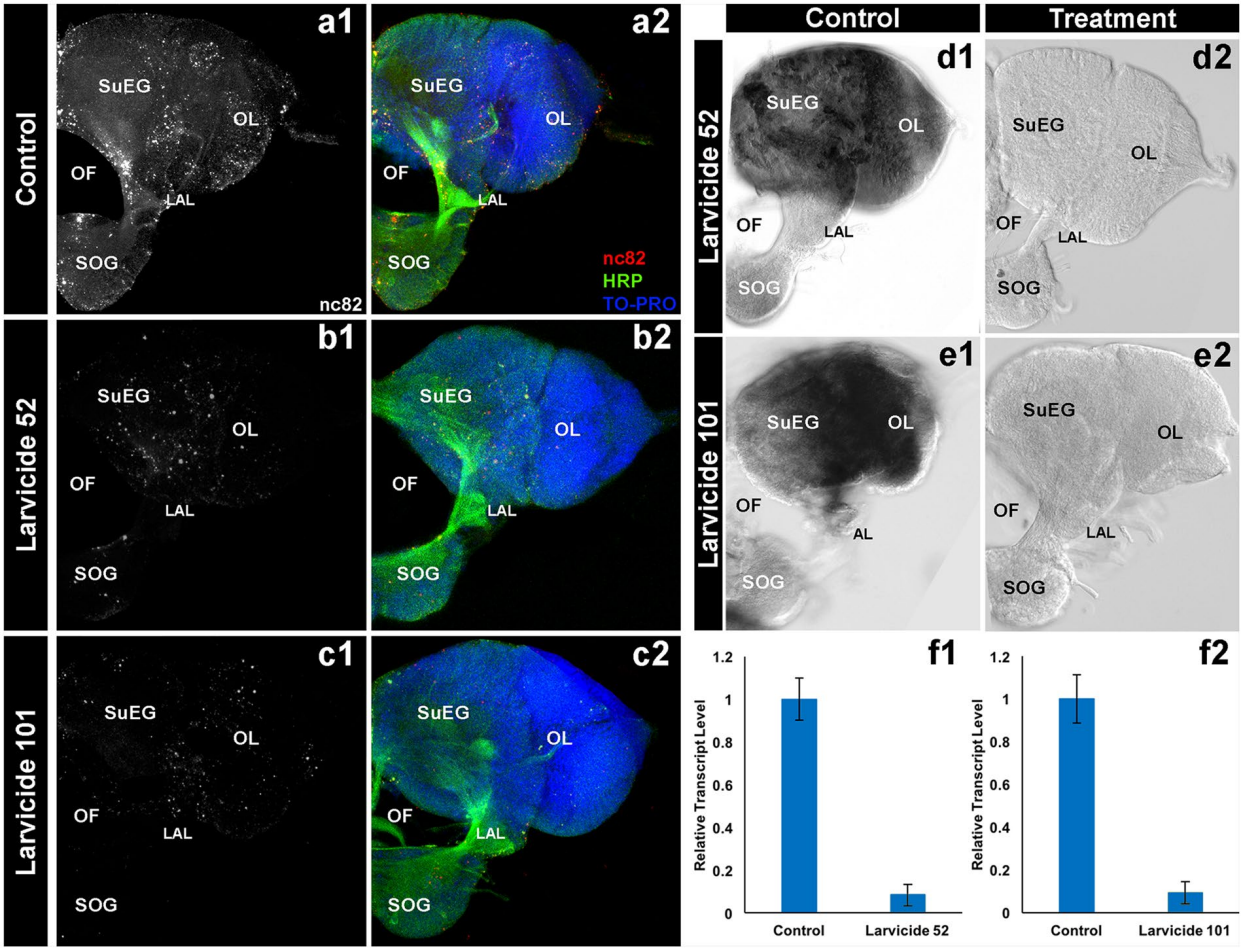
Neural defects observed in larvae treated with yeast interfering RNA larvicides #52 & #101. The brains of L4 *A*. *aegypti* larvae fed with dried inactivated yeast larvicide #52 and #101 vs. control tablets were labeled with mAbnc82 (white in **a1**, **b1**, **c1**; red in **a2**, **b2**, and **c2**) and HRP (green; a2, b2, c2) to visualize synaptic active zones and neurons, respectively. TO-PRO was used to counter-stain nuclei of the central nervous system (CNS; blue in a2, b2, c2). Although HRP levels are comparable, the brains of larvae fed with yeast larvicides #52 and #101 show loss of nc82 staining in the synaptic neuropile regions when compared with animals fed with control yeast (b1 and c1 vs. a1). These defects corresponded with significantly reduced transcripts of the #52 (**d2**) and #101 (**e2**) target genes in the L4 brain (compare to control expression levels of *fez2* and *lrc* transcripts, respectively in **d1** and **e1**). ***Significantly lower transcript levels were detected for larval brains fed with yeast larvicides #52 (**f1**) or #101 (**f2**) vs. animals fed with control yeast (P = 0.0001 in unpaired two tailed t-test in panel f1; P = 0.0001 in unpaired two tailed t-test in panel f2; error bars denote SEMs). For all of the experiments described in this figure, two biological replicate experiments, each with 20 animals in each of four replicate containers were assessed for each condition. LAL: Larval antennal lobe; OF: Olfactory foramen; OL: Optic lobe; SOG: Sub-oesophageal ganglion; SuEG: Supra-oesophageal ganglion. Brains are oriented dorsal upward in this figure.

Expression of *fez2* (Fig. 2d1) and *lrc* (Fig. 2e1) is detected in the fourth instar larval brain. Quantification of transcript levels in the larval brain confirmed significant silencing of both genes was achieved following larvicide ingestion, with 92 ± 5% reduction (P = 0.0001 vs. control yeast interfering RNA treatment) of *fez2* transcripts (Fig. 2d1,d2,f1) and 91 ± 5% reduction (P = 0.0001 vs. control yeast interfering RNA treatment) of *lrc* transcripts (Fig. 2e1,e2,f2) in brain tissues. HRP staining, which marks neurons, did not reveal significant differences in #52 (Fig. 2b2; P = 0.999) or #101 (Fig. 2c2; P = 0.3709) yeast-interfering RNA treated vs. control-treated (Fig. 2a2) L4 brains. However, expression of Bruchpilot (Brp), a marker of active zones in neural synapses that is labeled through nc82 antibody staining^21^, was significantly reduced in the synaptic neuropiles of L4 brains in animals fed with yeast interfering RNA larvicides #52 (Fig. 2b1,b2) or #101 (Fig. 2c1,c2; compare to controls in Fig. 2a1,a2). Quantification of nc82 staining levels in the L4 brain revealed a 97 ± 1% reduction of nc82 levels in #52-treated larvae (P = 0.0001 vs. control treatment) and 96 ± 1% reduction of nc82 levels in #101-treated larvae (P = 0.0001 vs. control treatment). Although the exact cause of death is unknown, these severe L4 neural phenotypes, which correlated with death of the larvae in L4 (Supplementary Fig. S2c,d), are likely primary causes of larval death in #52 and #101 larvicide-treated L4 animals.

As observed in *Drosophila* ^16^, loss of *fez2* results in mortality in late *A*. *aegypti* larval development. Although *Drosophila* larvae lacking function of this gene display obvious locomotor defects^16^, these were not obvious in *A*. *aegypti* larvae. However, it is possible that detailed tracing and analysis of larval swimming patterns could reveal more subtle defects that might be of higher consequence to wild mosquito larvae. It has been suggested that kinesin-mediated transport needs may increase in neurons during late larval development in flies, at which time both body size and neuron size increase rapidly. This may also explain why *fez2*-silenced *Aedes* larvae, like their *Drosophila unc-76* mutant counterparts^16^, die during late larval development, at which time *fez2*-silenced *A*. *aegypti* larvae display a lack of synaptic active zones in the brain, a defect which is presumably secondary to a more generalized loss of kinesin-mediated microtubule transport. Likewise, in the absence of *lrc*, which was predicted to function at the *Drosophila* synapse^20^, a lack of synaptic active zones in the L4 larval brain is observed. Proteins with the SAC3/GANP domain found in Lrc have a wide variety of cellular functions^16^, and it will be interesting to assess the precise roles of Lrc at the neural synapse in future studies. The *Drosophila* Dg-Dys complex, like its vertebrate counterparts, is believed to function in the transmission of information from the extracellular matrix to the cytoskeleton, and so Lrc may function in this capacity in the mosquito brain. Furthermore, given that it has been suggested that the *Drosophila* Dg-Dys complex interacts with Semaphorins^17^, it is interesting to note that a reduction in active zones as evidenced by decreased nc82 staining levels was also reported in *A*. *aegypti* larvae in which *semaphorin1a* had been silenced^5^.

### Preparation and characterization of yeast interfering RNA larvicides with integrated inducible shRNA expression cassettes

**Figure 3:**
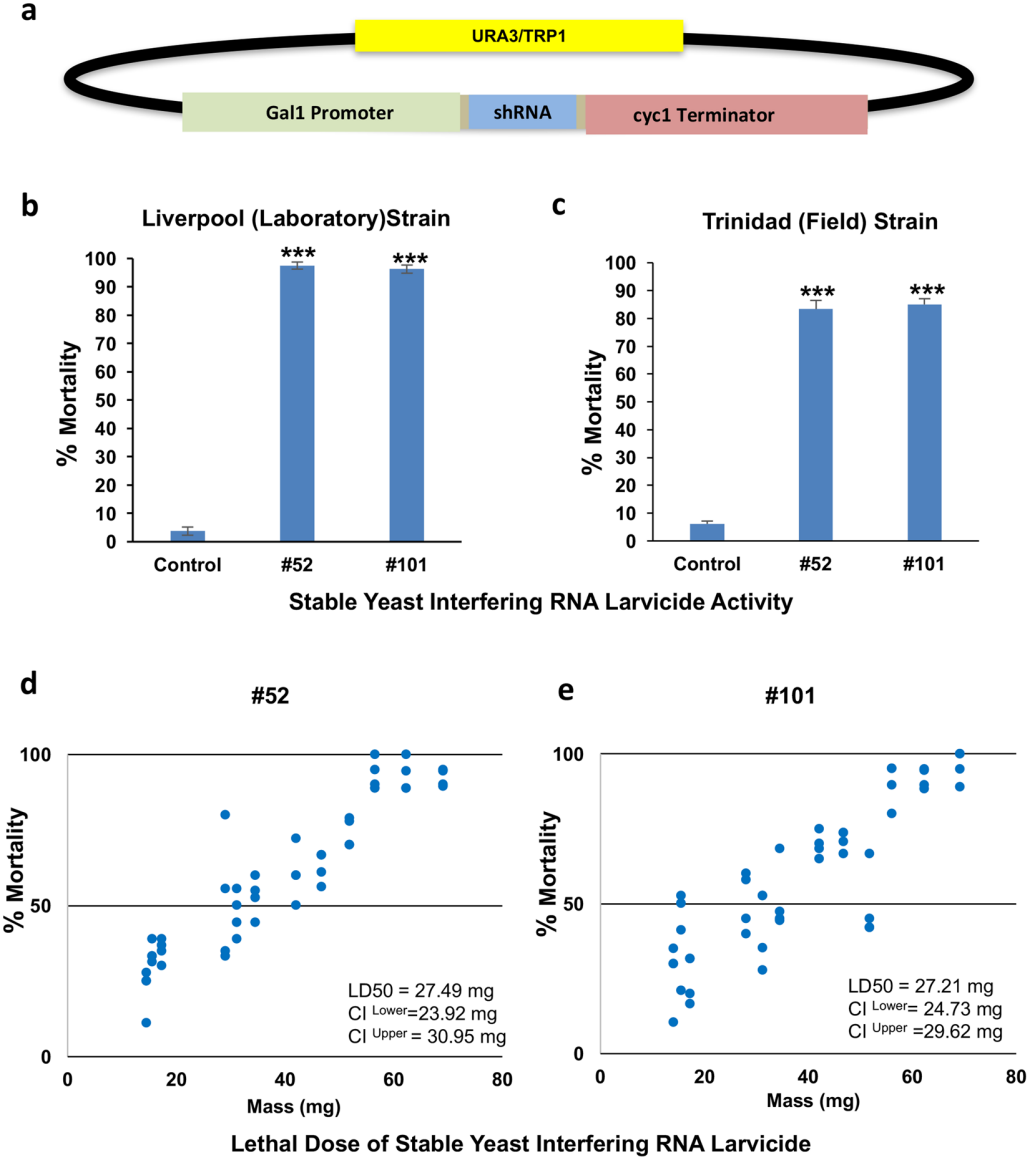
Generation of stably-transformed yeast interfering RNA larvicides that induce high rates of larval mortality. (**a**) Yeast integrating plasmid pRS404/406 constructs for integration of shRNA hairpin expression cassettes placed under control of the Gal1 galactose-inducible promoter were prepared and used to generate stable transformants. #52, #101, and control shRNA expression cassettes were integrated at both the *S*. *cerevisiae URA3* and *TRP1* loci. (**b**) Dry heat-inactivated tablets formed from strains with the #52 hairpin expression cassettes or the #101 hairpin cassettes integrated at both loci generated significant larval mortality in both Liverpool (**b**) and Trinidad (**c**) strain larvae, while animals fed with yeast expressing control shRNA lived. The data in b were compiled from two biological replicate experiments, each containing four replicates of 20 larvae, while data in c were compiled from three biological replicate experiments with three replicates of twenty larvae; the data are represented as % Mortality ± SEM. Data were analyzed by ANOVA with Tukey’s multiple comparison test (***P = 0.0001; significant differences exist between #52 vs. control-treated animals and #101 vs. control-treated animals). Dose-response curves depicting the mass of #52 (**d**) or #101 (**e**) stable yeast interfering larvicide vs. the percentage of Trinidad larval mortality are shown. LD50 values for #52 and #101 yeast interfering RNA larvicides are indicated. Further details regarding calculation of lethal doses are provided in the methods.

The results obtained with transiently transformed yeast suggest that yeast interfering RNA larvicides could be valuable tools for control of mosquitoes in the field (Fig. 1, Table 1). In preparation for field trials, #52, #101, and control shRNA expression cassettes were stably integrated at both the *TRP1* and *URA3* loci in the *S*. *cerevisiae* genome (Fig. 3a). Generation of these stable transformants eliminates the use of plasmids with antibiotic resistance markers and the potential for horizontal transfer of shRNA expression cassettes. In these strains, the #52, #101, and control shRNA expression cassettes were placed under control of an inducible Gal1 promoter (Fig. 3a), allowing for induction of shRNA expression through galactose inclusion in the media, followed by heat inactivation and harvesting of the yeast. Dry inactivated yeast tablets prepared from these stably transformed #52 and #101 yeast strains induced 97 ± 4% (P = 0.0001 vs. control yeast interfering RNA treatment) and 95 ± 5% (P = 0.0001 vs. control yeast interfering RNA treatment) mortality, respectively, in *A*. *aegypti* laboratory strain larvae (Fig. 3b). High levels of mortality were also induced following treatment of larvae hatched from the eggs of an *A*. *aegypti* strain recently established from mosquitoes collected in Trinidad (Fig. 3c). In Trinidad larvae, #52 and #101 yeast interfering RNA tablets prepared from stable yeast transformants induced 83 ± 3% mortality (Fig. 3d; p = 0.000001 vs. control treatment; LD50 = 27.49 mg) and 85 ± 2% mortality (Fig. 3e; p = 0.000001 vs. control treatment; LD50 = 27.21 mg), respectively.

Semi-field trials with these stably transformed inactivated yeast interfering RNA larvicides (Fig. 3) will demonstrate proof of concept of this new tool for mosquito control. These studies will evaluate efficacy, feasibility, as well as consumer attitudes toward the potential of introducing biorational yeast interfering RNA larvicides into integrated vector mosquito control programs. The field stability of yeast interfering RNA larvicides will of course need to be evaluated in these studies. However, dsRNA stability has been reported to be quite high^22,23^ and was found to be acceptable in our laboratory trials with heat-inactivated dried yeast tablets (Table 1c). Yeast interfering RNA larvicides that induce high levels of mortality in *Anopheles* mosquitoes have been identified (M. Duman-Scheel, unpublished). Thus, it is anticipated that the yeast interfering RNA larvicide approach could be extended to *Anopheles* mosquitoes and other mosquito vectors of disease. Furthermore, expression of dsRNA in *S*. *cerevisiae* was recently used to target genes in the agricultural pest *Drosophila suzukii* ^14^. Combined, these results suggest that yeast interfering RNA technology could potentially be used for biorational control of a wide variety of insect pests.

We anticipate that ready-to-use inactivated formulations of biorational yeast interfering RNA larvicides could seamlessly integrate, with minimal training and educational campaigns, into existing mosquito control programs to combat resistance to current pesticides and concerns for their potential impacts on non-target organisms. For example, *Bacillus thuringiensis israelensis* (Bti) and methoprene briquettes are currently used for treatment of *Aedes* breeding sites, as are granular formulations of temephos^24–27^. However, resistance to these pesticides has begun to emerge^28–34^. Furthermore, regulatory approval for temephos was not renewed in the United States last year^35^, and methoprene can be extremely toxic to non-target invertebrate species^25^. The results of this investigation demonstrate that dried inactive yeast interfering RNA tablets can function as lure-and-kill larvicides that offer biorational alternatives to these existing pesticides. Moreover, if resistance were to develop due to a mutation in any one shRNA target site in *A*. *aegypti*, an alternative target site could be used. Thus, by building an arsenal of different yeast interfering RNA larvicide strains, we can start to combat resistance before these larvicides are tested in the field. Baker’s yeast is manufactured in large-scale industrial cultures in a variety of active and inactive formulations that are readily packaged and shipped, making this technology versatile, scalable, and distributable. Moreover, yeasts have been cultivated worldwide for thousands of years, and this technology can therefore be adapted in resource-limited countries with constrained infrastructures.

## Methods

### Animal rearing

The *A*. *aegypti* Liverpool-IB12 (LVP-IB12) strain from which the genome sequence^36^ was generated was used in all the studies described, except for the trials described in Figs 3c–e and S2c, in which F6 animals of a strain recently established from eggs collected in ovitraps in Trinidad^37^ were used. Mosquitoes were maintained as described^38^, except that an artificial membrane feeding system was used for delivery of sheep blood (HemoStat Laboratories, Dixon, CA) to adult females. The mosquitoes were reared in an insectary at 26 °C, at ~80% humidity, and under a 12 hr light/12 hr dark cycle with 1 hr crepuscular periods at the beginning and end of each light cycle.

### Chitosan/siRNA nanoparticle, larval soaking, and microinjection experiments

Custom siRNAs corresponding to target sequences in *fez2* (*AAEL007292*), *lrc* (*AAEL007548*), and a control sequence with no known target in *A*. *aegypti* ^7^ were purchased from Integrated DNA Technologies (IDT). The target sequences of these siRNAs are:

#52*:* 5′CUAGCAUCAUCUUCCGACCGAACCA3′ in *fez2*,

#414: 5′CACUGACAAUGAUCCGAUAAAGACA3′ in *fez2*,

#418: 5′GUACCUAGUCGAUGGUCAAUCAGAG3′ in *fez2*,

#101: 5′GUAUCAGUCAGUAUCAGAACCAGAA3′ in *lrc*,

#467: 5′GCAGGAUUACUACUAUGCGUGUGAU3′ in *lrc*,

#468: 5′CGAAUGGAGUUUCAAGAUUCAUCGA3′ in *lrc*,

Control: 5′GAAGAGCACUGAUAGAUGUUAGCGU3′ (no known target in *A*. *aegypti*).

For testing of siRNA #52, chitosan/siRNA nanoparticles prepared with #52 or control siRNA were mixed with larval food and fed to 10 mosquito larvae/condition using previously described methods^8^. Larvae were then reared and assessed per World Health Organization (WHO) larvicide testing guidelines. Control vs. #52 treatment data were assessed with Fisher’s exact test. These experiments were part of a larger screen performed in duplicate (M. Duman-Scheel, unpublished) that will be described in its entirety elsewhere. Larval soaking experiments were performed in triplicate with 20 L1 larvae soaked at a concentration of 0.25 ug/ul for 4 hrs. with control vs. larvicide RNA per the methodology of Singh *et al*.^39^. For microinjection experiments, ~10 pmol custom screening siRNA were injected in a 30 nL volume per larva (n = 30/condition/replicate X two biological replicates) using previously described methodology^40,41^. Following siRNA treatment, larvae were reared and assessed per the WHO larvicide testing guidelines. Data were assessed using Fisher’s exact test. Soaking and microinjection experiments were performed in conjunction with larval lethal screens (M. Duman-Scheel, unpublished) that will be described in their entirety elsewhere.

### Bacteria interfering RNA larvicides

Heat-killed orally delivered non-pathogenic *E*. *coli* expressing dsRNA were constructed and fed to larvae beginning in the L1 stage as described by Whyard *et al*.^15^.

Strain *HT115-DE3*, obtained from the *Caenorhabditis* Genetics Center (CGC, which is funded by the NIH Office of Research Infrastructure Programs, P40 OD010440), was used. The strain was transformed with the dsRNA transcription plasmid pL4440 (deposited at Addgene by Andrew Fire; plasmid # 1654), which contains forward and reverse T7 polymerase binding sites that flank a multiple cloning site in which DNA corresponding to the siRNA #52 or #101 target sequences had been cloned. This allowed for inducible expression of dsRNA in bacteria, which were prepared and fed to larvae per the Whyard *et al*.^15^ protocol. *E*. *coli* transformed with GFP::L4440, which was deposited at Addgene by Guy Caldwell (plasmid # 11335), were used as a control feeding strain that expressed dsRNA corresponding to GFP. Larvicide assays were conducted per WHO guidelines. Seven replicate experiments, each with 20 larvae per treatment, were performed. The percentage of mortality was transformed to arcsine values for ANOVA comparisons (per WHO recommendations) followed by Tukey’s multiple comparison test.

### Generation of yeast interfering RNA larvicide strains

shRNA-encoding DNA oligonucleotides corresponding to the *A*. *aegypti* siRNA #52 target sequence in *fez2*, the siRNA #101 target sequence in *lrc*, and a control hairpin with no known *Aedes* target site^7^ were custom synthesized by Invitrogen Life Technologies. Two types of yeast transformants were generated:

#### Transient transformation

For transient transformation assays, the shRNA expression cassettes were cloned into *pRS426 GPD*, a non-integrating bacteria-yeast shuttle vector with a *URA3* marker that permits constitutive expression of inserts cloned downstream of a *GPD* promoter^42^. Following sequencing to confirm the inserts, the plasmids were transformed into *S*. *cerevisiae* strain *BY4742* ^43^, genotype *MATα his3Δ1 leu2Δ0 lys2Δ0 ura3Δ0*. Transformants were selected by growth on minimal media lacking uracil.

#### Generation of stable transformants

DNA encoding the #52, #101, or control shRNA was ligated downstream of the *Gal1* promoter^44,45^ and upstream of the *cyc1* terminator. The resulting *Gal1* promoter-shRNA-*cyc1* terminator expression cassettes were cloned into the multiple cloning sites of *pRS404* and *pRS406* ^46^, yeast integrating plasmid shuttle vectors bearing *TRP1* and *URA3* markers, respectively. The resulting plasmids were used for genome integration of the shRNA expression cassettes at the *trp1* and *ura3* loci of the *S*. *cerevisiae CEN*.*PK* strain (genotype = *MAT*a/α *ura3-52/ura3-52 trp1-289/trp1-289 leu2-3_112/leu2-3_112 his3* Δ*1/his3* Δ*1 MAL2-8C/MAL2-8C SUC2/SUC2*)^47^. Stable transformants were selected by growth on synthetic complete media lacking tryptophan or uracil. Integration events at both loci were confirmed via PCR and sequencing.

### Yeast culturing

Following selection as described above, yeasts were grown under standard conditions in synthetic media to an OD_600_ of 3.0. For galactose induction experiments, yeasts were cultured in 20 ml SCD medium containing 20 g/L glucose to early stationary growth phase. Cells were harvested by centrifugation and transferred into 200 ml of fresh SCD medium containing 20 g/L galactose along with 2 g/L glucose. This was cultured at 30 °C and 250 rpm for 18 h (OD600 ~ 3.0).

### Preparation of yeast larvicide formulations

For initial experiments, yeast-agarose tablets were prepared for feeding assays from 50 ml of liquid yeast culture as described in the Whyard *et al*.^15^ procedure for production of both live and heat-inactivated bacterial pellets. For preparation of dried tablet formulations, yeast cultures were grown as indicated above. The culture was then transferred to 50 ml conical tubes, each containing 50 ml of culture for transient transformants or 40 ml of culture for stable transformants, and pelleted by centrifugation for 20 min at 4000 rpm (Eppendorf, 5810 R plus). The supernatant (media) was discarded before placing the pellet in a 70 °C water bath for 5 mins. The yeast pellet was then placed into a 2 ml tube containing 10 mg of liver powder (MP Biomedicals, LLC, Solon, OH) as a nutritional supplement and centrifuged for 1 min at ~13.2 rpm (Eppendorf, 5415D). The supernatant was again discarded, and the tubes were left open in an incubator at 30 °C for 48 hrs to evaporate remaining media. The tablets were allowed to dry before storage in capped microfuge tubes at −20 °C. The final weight of each tablet averaged 95 mg for transiently-transformed yeast and 82 mg for stable yeast transformant tablets, each including 85 mg and 72 mg of yeast (~1.6 × 10^10^ cells) respectively, as well as 10 mg of liver powder.

### Larval feedings with yeast

Agarose gel coated formulations were divided into three portions, with one portion fed daily for three days to 20 first instar larvae (L1) per replicate experiment. For dried tabular formulations, one control or experimental tablet was placed in each container with 20 L1 animals at the start of each experiment. Larval bioassays were performed in 500 ml plastic cups containing 50 ml of distilled water and conformed to the WHO larvicide testing guidelines^48^. Larvae fed with larvicides #52, #101, and the control larvicide formulations were evaluated in parallel in at least three biological replicate experiments, each with at least three replicates per condition. The ages of larvae (days post-hatching and larval instar) at the time of death were recorded. Body lengths of control and larvicide-treated animals were also assessed using Fiji ImageJ software by measuring the distance between the head to the 8^th^ abdominal segment in treated early L4 larvae that had been fixed and photographed; body length data were statistically analyzed with ANOVA. Dried tablet formulations were also assessed in 33 cm × 23 cm × 5 cm aluminum pans and 18.9 L white buckets containing 1.5 L and 15.75 L of distilled water, respectively and evaluated in parallel in three biological replicate experiments, with three replicates in 0.050 L water, and two replicates in 1.5 L and 15.75 L water. For statistical analysis of larval mortality assays, the percentage of mortality was transformed to arcsine values for ANOVA (per the WHO^48^ recommendations) followed by Tukey’s multiple comparison test.

### Lethal dose determination

Initially, mosquitoes were exposed to various amounts of control, #52, and #101 yeast to determine the activity range. Control yeast was mixed with #52 and #101 yeast to various concentrations to generate the dried inactivated yeast tablets. First instar larvae were then fed and assessed as described above. Four and three biological replicate experiments with at least four replicate containers per concentration were run for #52 and #101, respectively. Abbot’s formula was used to account for mortality in control animals, as discussed in the WHO guidelines^48^. Data from all replicates were pooled for analysis. LD50 with 95% confidence intervals were calculated from a log dosage-probit mortality regression line using SPSS computer software.

### Assessment of residual activity

Following preparation of dried inactivated yeast tablets prepared using the aforementioned protocol, the tablets were placed in 50 ml conical tubes, flooded with 50 ml of distilled water, and used immediately or stored in the insectary for one to two weeks prior to use. Prior to larval feeding, the yeast and water mixture was transferred into plastic cups and allowed to precipitate (for ~5 hours) before adding the larvae, which were then assessed per WHO guidelines^48^. Larvae fed with larvicides #52, #101, and the control larvicide formulations were evaluated in parallel in three biological replicate experiments with three replicates for each week. The weekly percentages of mortality were transformed to arcsine values for ANOVA (per WHO^48^) followed by Tukey’s multiple comparison test.

### Whole mount *in situ* hybridization and immunohistochemistry

Riboprobes corresponding to *fez2* and *lrc* were synthesized according to the Patel^49^ protocol, and *in situ* hybridization experiments were performed in duplicate as previously described^50^. Living animals were fixed for these assays. Tissues were then mounted and imaged with a Zeiss Axioimager equipped with a Spot Flex camera. For transcript quantification analyses, mean gray values (average signal intensity over the selected area) were calculated for digoxigenin-labeled transcript signal in control or experimental brains combined from two biological replicate experiments, each with 20 animals in each of four replicate containers for each condition. Transcript quantification data were statistically analyzed using an unpaired two tailed t-test. Immunohistochemical staining experiments were performed as previously described^51,52^ using mAb nc82 anti-Bruchpilot^21^ (DSHB Hybridoma Product nc82, which was deposited to the DSHB by E. Buchner), anti-HRP (Jackson Immunoresearch, West Grove, PA) and TO-PRO-3 iodide (Molecular Probes, Eugene, OR). Two biological replicate experiments, each with 20 animals in each of four replicate containers per condition, were processed. Living larvae were fixed for these experiments. After processing, tissues were mounted and imaged on a Zeiss 710 confocal microscope using Zen software. Images were analyzed with FIJI ImageJ and Adobe Photoshop CC 2014 software. For antibody staining intensity analyses, mean gray values were calculated as described^6^ for control or experimental brains combined from two replicate experiments. Data were statistically analyzed using one-way ANOVA followed by the Kruskal-Wallis post hoc test.

### Oviposition response assays

The impact of yeast interfering RNA larvicides on the attraction of gravid females was assessed. Heat-inactivated yeast was evaluated in these trials, which were conducted in laboratory assays performed in the insectary. Oviposition was measured with 10 gravid females released in a 30 cm × 30 cm × 30 cm cage containing two ovicups. Ovicups consisted of a 300 ml plastic cup lined with white paper towel filled with 100 ml of rainwater with and without a dried yeast tablet containing heat-inactivated control interfering yeast larvicide. Eggs were collected for four days during each assay. Four biological replicate experiments, each with five cage replicates, were assessed. Data were evaluated with a paired two tailed t-test. After a four-day egg laying period, a paired two tailed t-test was used to compare the number of eggs laid in the two treatments (4 replications and 5 repetitions, n = 20).

### Ethics statement

No human subjects or vertebrate animals were used in this investigation.

### Data availability

All data generated or analyzed during this study are included in this article.

## Electronic supplementary material

Supplementary Figures
