# Supplementary material for: Lure-and-Kill Yeast Interfering RNA Larvicides Targeting Neural Genes in the Human Disease Vector Mosquito Aedes aegypti
Source: Sci Rep. 2017 Oct 16;7:13223. doi: 10.1038/s41598-017-13566-y (PMC5643370; doi:10.1038/s41598-017-13566-y)
Supplement: Supplementary file 1 — Supplementary Figures [file 41598_2017_13566_MOESM1_ESM.pdf]

Lure-and-Kill Yeast Interfering RNA Larvicides Targeting Neural Genes in the Human Disease Vector Mosquito *Aedes aegypti*

Limb K. Hapairai,[1,2,Ψ] Keshava Mysore,[1,2,Ψ] Yingying Chen,[2,4] Elizabeth I. Harper,[1,2] Max P. Scheel,[1] Alexandra M. Lesnik,[2] Longhua Sun,[2,3] David W. Severson,[1,2,3] Na Wei,[2,4] and Molly Duman-Scheel,[1,2,3,*]

1) Indiana University School of Medicine, Department of Medical and Molecular Genetics, South Bend, IN, USA

2) The University of Notre Dame Eck Institute for Global Health, Notre Dame, IN, USA

3) The University of Notre Dame Department of Biological Sciences, Notre Dame, IN, USA

4) The University of Notre Dame Department of Civil and Environmental Engineering, Notre Dame, IN, USA

Ψ These authors contributed equally to this work

*Contact PI:
Molly Duman-Scheel
mscheel@nd.edu
Raclin-Carmichael Hall
1234 Notre Dame Ave.
South Bend, IN 46617
(574) 631-7194

a siRNA #52 target site: corresponds to bases 95-119 of *fez2* (*AAEL007292)* exon 1

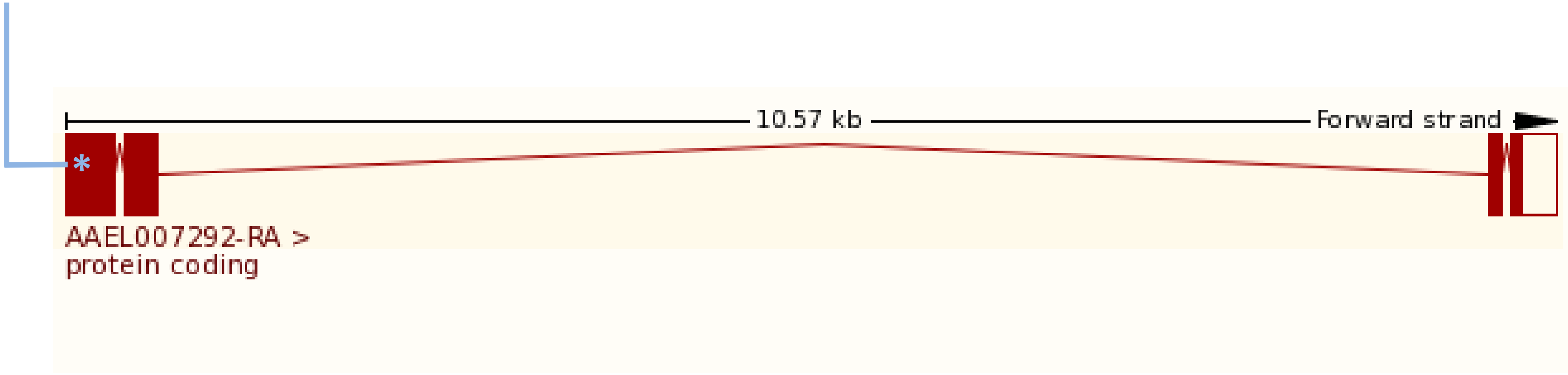

b siRNA #101 target site: corresponds to bases 473-497 of *lrc (AAEL007548)* exon 1

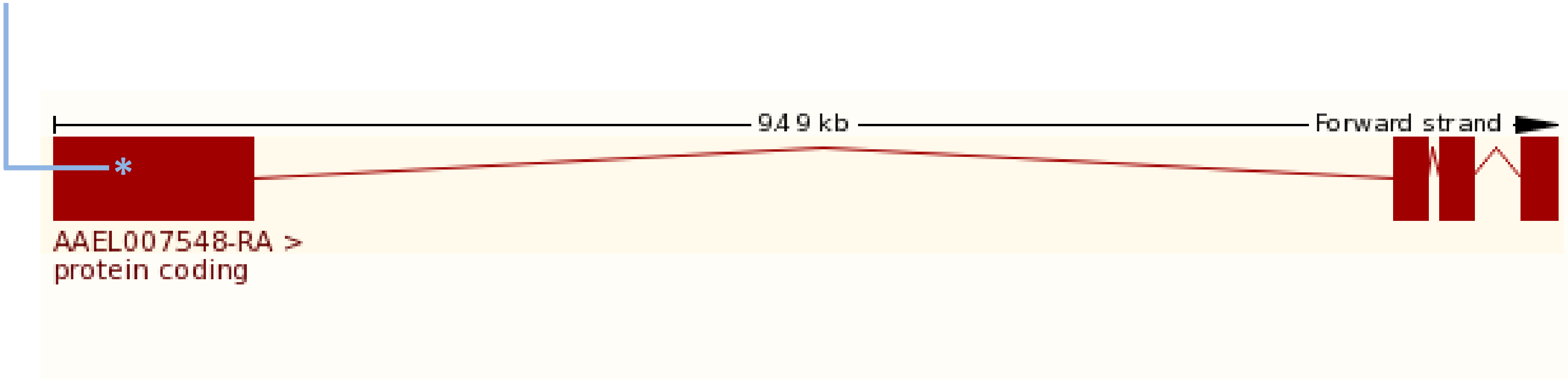

Supplementary Fig. S1. Target sites of siRNAs #52 and #101. The target sites (marked by *) of siRNA/shRNA #52 (a) and #101 (b) correspond to sequences in the first exons of *fez2* (a) and *lrc* (b). Exons are shown as boxes, with filled boxes denoting coding regions. Exon/intron structure images were exported from VectorBase[11].

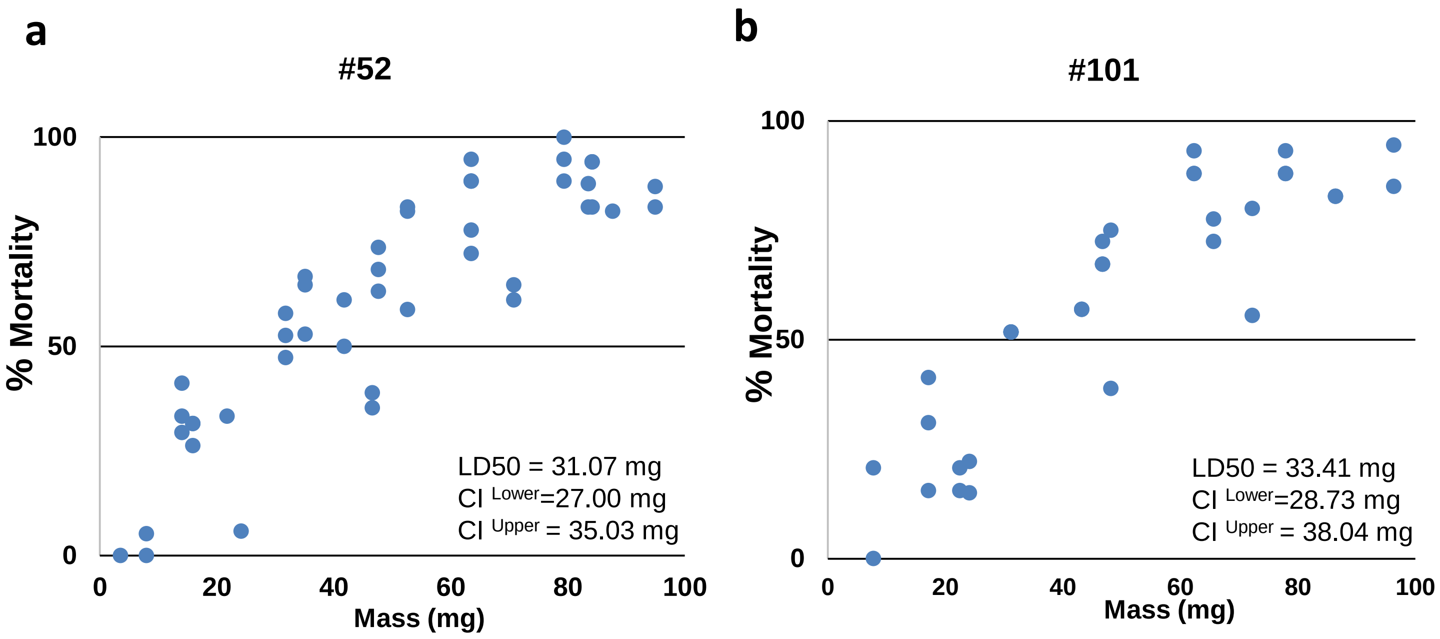

Lethal Dose of Yeast Interfering RNA Larvicides

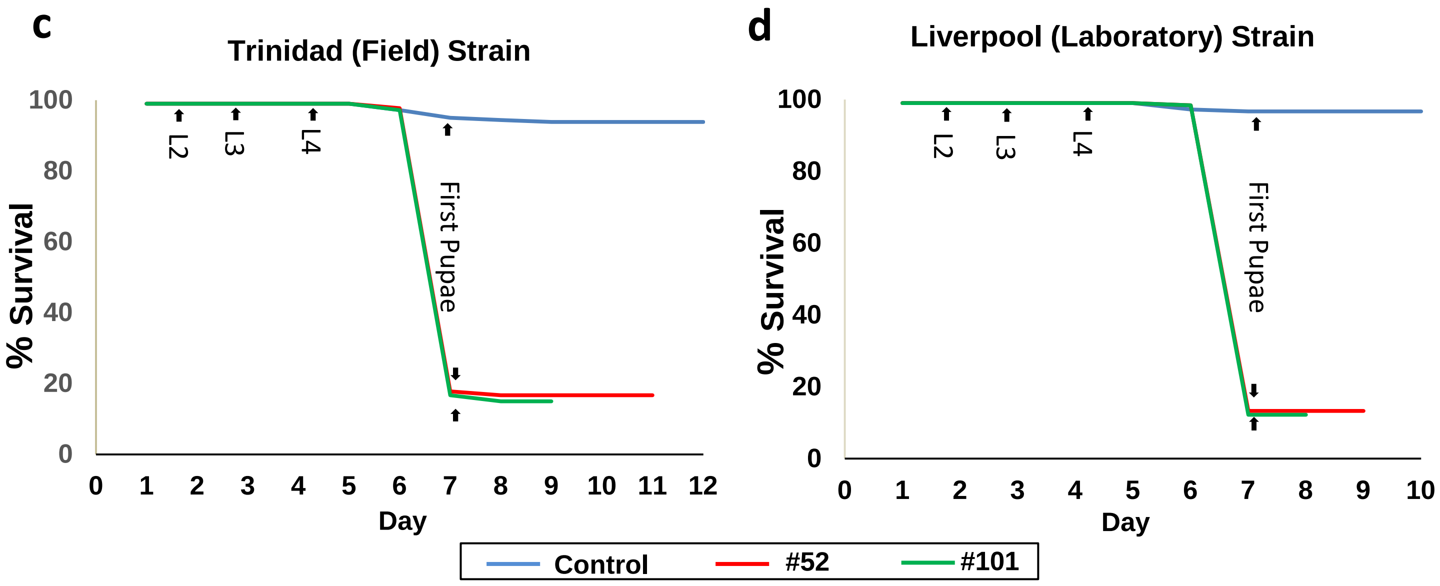

Survival Curves following Treatments with Yeast Interfering RNA Larvicides

Supplementary Fig. S2. The impacts of yeast interfering RNA larvicides #52 and #101 on larval survival. Dose-response curves depicting the mass of transiently-transformed yeast interfering RNA larvicides #52 (a) and #101 (b) vs. larval mortality are shown; LD50 values with upper and lower confidence limits (CL) for the yeast interfering RNA larvicides are indicated. Further details regarding calculation of lethal doses is provided in the methods. The daily percentages of survival post-larvicide treatment of Trinidad (c) and Liverpool (d) strain larvae are indicated. The experiment shown in c was performed with stable transformant yeast interfering RNA larvicide tablets, while transiently transformed yeast interfering RNA larvicide tablets were used in d. Larvae treated with yeast interfering RNA larvicides #52 and #101 die in L4, with the majority of animals failing to pupate.
